# A New Tessera into the Interactome of the *isc* Operon: A Novel Interaction between HscB and IscS

**DOI:** 10.3389/fmolb.2016.00048

**Published:** 2016-09-27

**Authors:** Rita Puglisi, Robert Yan, Salvatore Adinolfi, Annalisa Pastore

**Affiliations:** ^1^Department of Basic and Clinical Neuroscience, Maurice Wohl Institute, King's College LondonLondon, UK; ^2^Molecular Medicine Department, University of PaviaPavia, Italy

**Keywords:** biogenesis, hybrid methods, integrative biology, interactome, iron-sulfur clusters

## Abstract

Iron sulfur clusters are essential universal prosthetic groups which can be formed inorganically but, in biology, are bound to proteins and produced enzymatically. Most of the components of the machine that produces the clusters are conserved throughout evolution. In bacteria, they are encoded in the *isc* operon. Previous reports provide information on the role of specific components but a clear picture of how the whole machine works is still missing. We have carried out a study of the effects of the co-chaperone HscB from the model system *E. coli*. We document a previously undetected weak interaction between the chaperone HscB and the desulfurase IscS, one of the two main players of the machine. The binding site involves a region of HscB in the longer stem of the approximately L-shaped molecule, whereas the interacting surface of IscS overlaps with the surface previously involved in binding other proteins, such as ferredoxin and frataxin. Our findings provide an entirely new perspective to our comprehension of the role of HscB and propose this protein as a component of the IscS complex.

## Introduction

Iron–sulfur (FeS) clusters are essential acid-labile prosthetic groups containing iron and sulfur which act as electron transfer agents thanks to their versatile electron-donor/acceptor properties (Beinert et al., 1997). In nature, they are widely distributed in most organisms, including anaerobic, aerobic and photosynthetic bacteria, fungi, plants, and animals (Mansy and Cowan, 2004). FeS clusters can be chemically assembled on proteins from the required components under reducing conditions or, more efficiently, produced enzymatically (Bonomi et al., 1985). Investigation on FeS cluster maturation in bacteria has led to the identification of up to three operons which encode most of the components required for enzymatic FeS cluster assembly. Among these, the *isc* operon is the most universal, being activated in exponentially growing cells (Py et al., 2011) and having high sequence similarity to the eukaryotic components of FeS protein maturation (Lill and Kispal, 2000). Given the high degree of homology with the eukaryotic system, a detailed knowledge of this machine has potential relevance for human iron-storage diseases. Abnormal FeS protein biogenesis and mitochondrial iron accumulations are for instance part of the typical phenotype of an increasing number of genetic diseases including Friedreich's ataxia (Puccio and Koenig, 2000; Pandolfo and Pastore, 2009), hereditary IscU myopathy with lactic acidosis (Olsson et al., 2008) and combined oxidative phosphorylation deficiency-19 (Lim et al., 2013).

In *E. coli*, the *isc* operon encodes eight proteins: IscR, IscS, IscU, IscA, HscB, HscA, Fdx, and YfhJ (Tokumoto and Takahashi, 2001). Strains with mutations in the *iscU, hscB, hscA*, and *fdx* genes exhibit conspicuous phenotypical consequences almost identical to one another. The two central proteins of the machine are IscS and IscU (Nfs1-Isu in eukaryotes; Smith et al., 2001). IscS is a pyridoxal phosphate (PLP) desulfurase which converts cysteine into alanine and releases a reactive persulfide (Zheng et al., 1993). IscU is the scaffold protein which transiently hosts the FeS cluster before this is transferred to other acceptors (Agar et al., 2000). They work together in a 1:1 complex where two copies of IscU bind to the obligate dimer of IscS on binding sites very distant from each other (Prischi et al., 2010a; Shi et al., 2010). Amongst the other components are the DNA binding protein and operon regulator IscR (Schwartz et al., 2001; Fleischhacker et al., 2012), the alternative scaffold protein IscA (Krebs et al., 2001; Ollagnier-de-Choudens et al., 2001; Bonomi et al., 2005), a ferredoxin (Fdx) which provides electrons to the reaction (Kim et al., 2013; Yan et al., 2013, 2015), YfhJ, a protein of unknown function absent in most eukaryotes (Shimomura et al., 2005; Pastore et al., 2006), and the proteins HscA and HscB.

The role of the latters are somewhat contradictory: based on their homology with DnaK and DnaJ (Vickery et al., 1997), they are chaperones thought to assist cluster transfer (Hoff et al., 2000; Chandramouli and Johnson, 2006). Nevertheless, their presence was recently found to slow down rather than facilitate the reaction both in the enzymatic and in the non-enzymatic reactions (Iametti et al., 2015). This finding was explained by assuming that interactions with IscU, which is known to be a partner of both HscB and HscA (Hoff et al., 2000), could interfere with the process of cluster transfer to another acceptor. While possible in principle, the result strongly demands further investigations which could clarify the role of these proteins in the *isc* machine and, more generally, of chaperones. A way forward to shed light on this important point is to understand further the complex network of interactions among the *isc* proteins by dissecting the complete interactome of the *isc* machine which is at the moment far from being complete.

Here, we studied the role of HscB in cluster transfer using a hybrid methodology based on a combination of NMR, biophysical methods and site directed mutagenesis. We first screened the multidimensional space of the relative molar ratios not only of the desulfurase IscS and scaffold IscU, but also of HscA and HscB to understand how this parameter affects the machine functioning. We then concentrated on HscB since this protein has been suggested to be the hub between the scaffold IscU and HscA. Quite surprisingly, we observed and characterized a novel interaction between the co-chaperone HscB and the desulfurase IscS. HscB does not displace IscU but competes for the same site of IscS which accommodates Fdx (Yan et al., 2013), YfhJ (Pastore et al., 2006), and frataxin (Prischi et al., 2010b), the protein responsible for Friedreich's ataxia. The interaction is weak but comparable to that of other components suggesting that the competition constitutes the basis of the machine regulation. These results lead to a complete shift of our understanding of the iron sulfur cluster machine: IscS/IscU and the chaperones may act in a concerted way and form a unique complex.

## Results

### Optimization of IscU, HscA, HscB, and ATP concentrations

Enzyme activity was measured using the *E. coli isc* proteins IscS, IscU, HscA, HscB, and apo Fdx. This *in vitro* assay is a sensitive method to follow the enzymatic rates by detecting the absorbance of a reporter acceptor as a function of time (Adinolfi et al., 2009; Prischi et al., 2010b). We monitored the signal at 458 nm because this is the characteristic wavelength for the absorbance of FeS clusters on Fdx. Because we work with a complex multi-component system, we first optimized the relative concentrations of the components. The concentration of apo Fdx, which was used as the final cluster acceptor, was set to 50 μM which is a value that allows easy measurement of the absorbance signal from the cluster. IscS was set to catalytic concentration (1 μM) to minimize contributions to the spectrum of unspecific iron-thiolate polysulfides bound to IscS (Bonomi et al., 1985). The presence of the antioxidant DTT is necessary to provide the reducing equivalents required for cluster generation and to regenerate the prosthetic group pyridoxal phosphate (Urbina et al., 2001). We started with 1 μM IscU, 1 μM HscA, and 1 μM HscB, 250 μM Cys, 150 μM ATP, 25 μM Fe^2+^ and, unless explicitely indicated, 10 mM Mg^2+^. We measured the slope of the curves after the lag phase when present to compare the rates semi-quantitatively.

**Figure 1:**
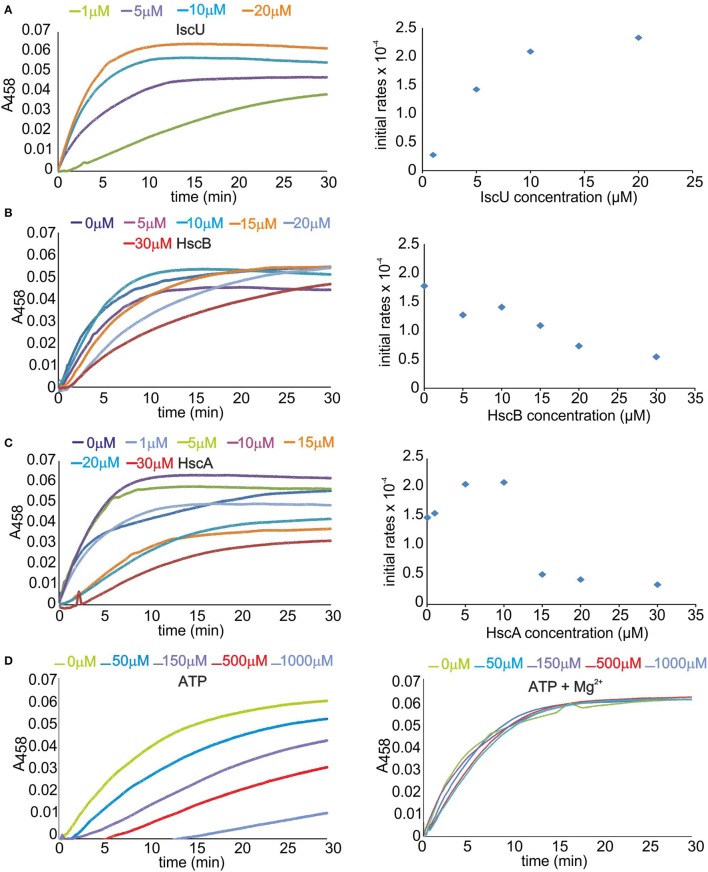
**Optimizing the concentrations of the Isc components on the formation of the cluster on Fdx**. **(A)** Left: Scan of the IscU concentration. Rates of cluster formation on 50 μM Fdx in the presence of 1 μM IscS, 1 μM HscA, 1 μM HscB, 3 mM DTT, 250 μM cys, 150 μM ATP, 25 μM Fe^2+^, and 10 μM Mg^2+^. IscU was progressively 1 μM (green), 5 μM (violet), 10 μM (cyan), and 20 μM (orange). Right: Corresponding rates estimated from the slope of the initial part of the curves after the lag time. **(B)** Left: Scan of the HscB concentration. The concentrations of IscU and HscA are fixed at 8 and 2 μM. HscB is varied from 0 μM (blue), 5 μM (violet), 10 μM (cyan), 15 μM (orange), 20 μM (pale blue), and 30 μM (red). The other components are as in the previous experiment. Right: Corresponding initial rates. **(C)** Left: Scan of the HscA concentration. The concentrations of IscU and HscB are fixed to 8 and 1 μM. The other components are as above. HscA was varied from 0 μM (blue) to 1 μM (pale blue), 5 μM (green), 10 μM (violet), 15 μM (orange), 20 μM (cyan), and 30 μM (red). Right: Corresponding initial rates. **(D)** Left: Scan of the ATP concentration in the absence of Mg^2+^. IscU, HscA, and HscB are fixed at 8, 2, and 3 μM, respectively. ATP is varied from 0 μM (green) to 50 μM (cyan), 150 μM (violet), 500 μM (red), and 1 mM (pale blue). Right: The same as the left panel but with the addition of 10 mM Mg^2+^.

Increasing IscU concentrations (1, 5, 10, 20 μM) enhanced the reconstitution rate and shortened the lag phase, reaching a plateau above 5–10 μM (Figure 1A). This is reasonable since increase of IscU concentration facilitates formation of the IscS-IscU complex and accelerates the reaction. We thus adopted 8 μM concentrations of IscU in the following experiments to ensure high activity but, at the same time, avoid contributions from holo-IscU to absorbance. Progressive increase of HscB concentration caused a clear reduction of the reaction rates (Figure 1B). A 3 μM HscB concentration was adopted in the following experiments to ensure a small excess of protein with respect to IscS in a range of concentrations where inhibition is not dominant. The ATPase activity of HscA was independently checked by a spectrophotometric method that allows the quantification of inorganic phosphate release (data not shown). Variation of the HscA concentration in the range 1–5 μM led to no change of the cluster formation rates. Above ~5 μM, we observed a deep decrease of the rates (Figure 1C). A 2 μM concentration was thus adopted in the following measurement to ensure a small excess of protein with respect to IscS. Finally, we screened the effect of ATP varying it from 0 to 1 mM in the absence and in the presence of 10 mM Mg^2+^. When no Mg^2+^ was added we observed a dramatic reduction of the rates up to almost complete inhibition of the reaction (Figure 1D). The effect was drastically reduced and practically abolished at 10 mM Mg^2+^ which are the concentrations typically used for this assay because close to the cellular conditions.

These results reproduce a previous study which was however carried out mainly in the absence of IscS (Iametti et al., 2015). A plausible way to explain the effect of ATP, which had been left with no explanation, is that ATP is well known to chelate iron (Mansour et al., 1985; Patchornik et al., 2000). It would then result in depletion of Fe^2+^ from solution. Mg^2+^, the counter ion always used with chaperones, is thus particularly important to compete with Fe^2+^ binding.

### Dissecting the determinants of the effects on enzymatic activity

**Figure 2:**
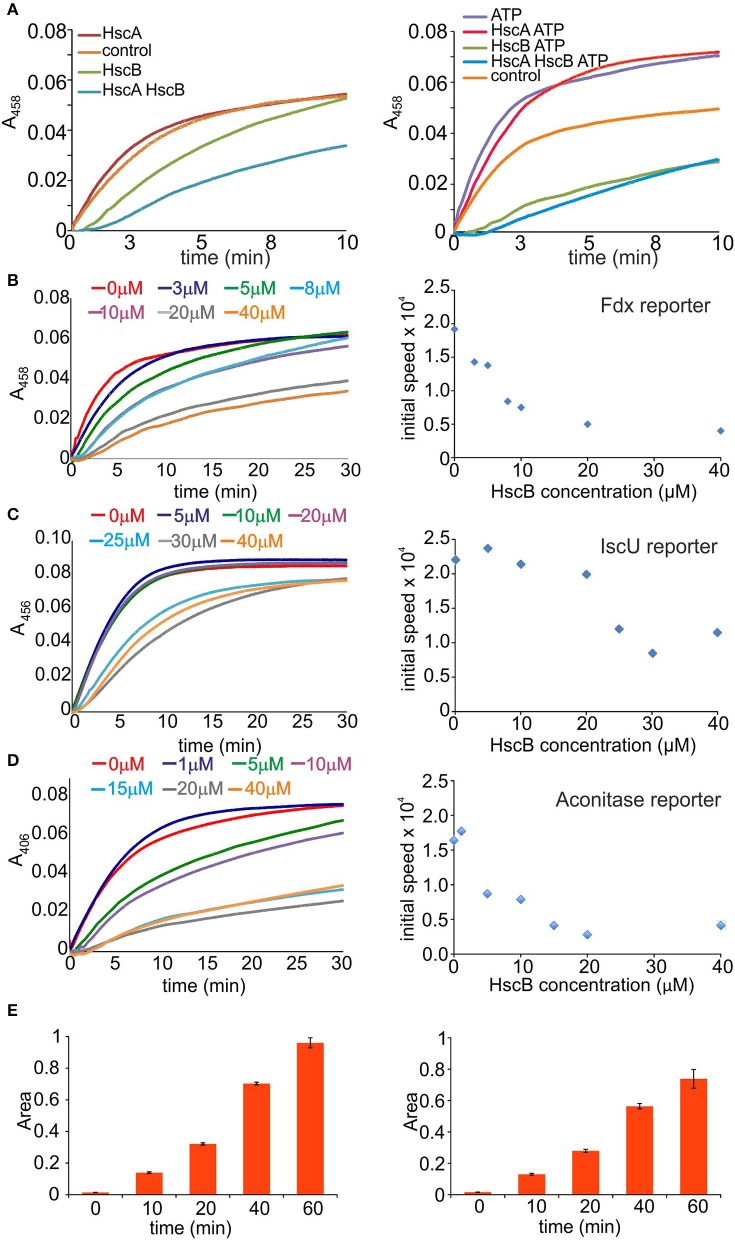
**Identification of the component(s) responsible for slowing down the reaction. (A)** Effect of the individual components. Cluster formation rates on Fdx in the presence of IscS and IscU (control, orange), HscA (red), HscB (green), or HscA/HscB (cyan). The experiment is repeated in the absence (left) and presence of ATP (right). **(B)** Time course of cluster formation on Fdx in the presence of IscS, IscU, DTT with Cys and Fe^2+^ in the presence of increasing concentrations of HscB. The curves correspond to no HscB (red), 3 μM (blue), 5 μM (green), 8 μM (cyan), 10 μM (purple), 20 μM (gray), and 40 μM (orange). Right: Corresponding rates. **(C)** Cluster formation rates on IscU in the presence of IscS, DTT with Cys, and Fe^2+^. The curves correspond to no HscB (red), HscB 5 μM (blue), 10 μM (green), 20 μM (purple), 25 μM (cyan), 30 μM (gray), and 40 μM (orange). Right: Corresponding rates. **(D)** Cluster formation rates on aconitase in the presence of IscS, IscU, DTT with Cys, Fe^2+^. The curves correspond to no HscB (red) and HscB 1 μM (blue), 5 μM (green), 10 μM (purple), 15 μM (cyan), 20 μM (gray), and 40 μM (orange). Right: Corresponding rates. **(E)** Quantification of alanine formation. Alanine production by IscS (left) and in the presence of HscB (right).

To understand the factors which determine inhibition of HscB and HscA, we explored the effects of each component by introducing them individually and in pair in the absence and in the presence of ATP (Figure 2A). We used again Fdx as the reporter, 1 μM of IscS and 8 μM of IscU, in the presence of 250 μM Cys, 25 μM Fe^2+^, and 10 mM Mg^2+^. In the absence of ATP, introduction of HscA (10 μM) does not produce appreciable effects on the enzymatic rates. Addition of HscB (10 μM) remarkably inhibits the rates, while co-addition of HscA and HscB produces a further drop. In the presence of ATP, addition of HscA increases the rates in a comparable way than when adding only ATP. Addition of HscB, alone or together with HscA and ATP appreciably reduces the rates. This tells us that the effect is mainly due to HscB, whether in the presence or absence of HscA.

We thus decided to focus on HscB and leave for future studies the more complex effect of HscA, which is further complicated by ATP. We first repeated the screening of HscB concentrations, but this time in the absence of ATP and HscA. We observed a clear progressive drop of the rates (Figure 2B left panel). Plotting the slope of the curves vs. the concentration we obtained a rate decrease, which reaches a plateau between 20 and 40 μM (Figure 2B right panel). We wondered if the effect could be due to the protein reporter given that Fdx is somewhat a special case because it interacts specifically with IscS (Yan et al., 2013). We thus repeated the measurements using IscU or aconitase (Beinert et al., 1996) as reporter (35 μM). Addition of HscB decreased both rates as in the previous cases (Figures 2C,D). The effect is thus independent from the reporter.

We then measured the desulfurase activity by detecting the progressive disappearance of cysteine and concomitant appearance of alanine by mass spectrometry in the absence of IscU at different time points which allowed us to follow the rates of desulfuration. We used the same concentrations as in the previous assays. Once initiating the reaction by adding the substrate, mixtures of acetic acid/acetonitrile were used to stop it. The presence of HscB slows down the reaction resulting in slower rates (Figure 2E).

Altogether, these results tell us that the inhibitory effect is mainly linked to HscB but it is not only due to the interaction between IscU and HscB as previously suggested (Hoff et al., 2000; Iametti et al., 2015): HscB has also effects on the desulfurase step and thus on IscS.

### HscB binds IscS with micromolar affinity through the protein C-terminus

**Figure 3:**
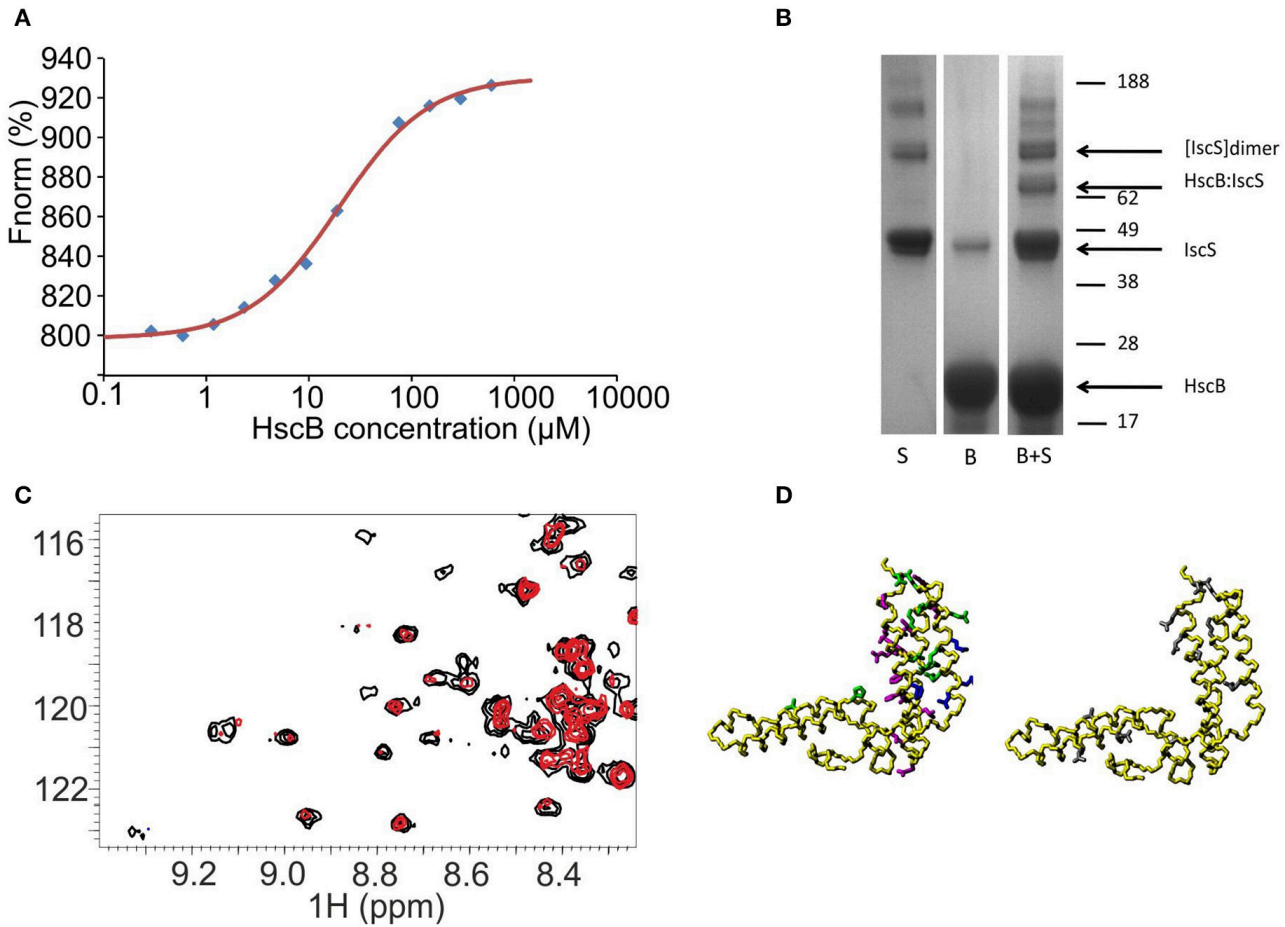
**Probing a direct interaction between IscS and HscB**. **(A)** MST data for the IscS and HscB interaction. Data are plotted as normalized signal changes as a function of the HscB concentration. The average of three experiments is reported. **(B)** SDS-PAGE of IscS, HscB, and IscS:HscB mixture in the presence of a cross-linker (BS3). **(C)** HSQC-NMR spectra of 15N-labeled HscB 100 μM (black) and 15N-labeled HscB 100 μM in the presence of IscS 1:1 (red). **(D)** Mapping the interaction surface on HscB (PDB code: 1FPO). Left: the residues showing chemical shift perturbation at a 1:1 molar ratio are shown in green, those which broaden or disappear in magenta. For comparison, the residues identified as involved in IscU binding are shown in blue. Right: residues giving cross-saturation with IscS at the same molar ratio (gray).

Although not previously reported, a possible working hypothesis to explain the observed effect is that HscB binds also to IscS and that this interferes with the desulfuration step. To test the hypothesis, we carried out pull-down and size exclusion chromatography assays but both proved inconclusive, as it can be expected for weak complexes. We thus used microscale thermophoresis (MST) which is a sensitive means to measure weak interactions (Jerabek-Willemsen et al., 2014). The thermophoretic movement of the fluorescently labeled IscS was assessed by measuring the fluorescence distribution inside a capillary and the microscopic temperature gradient generated by a laser. The binding curve was obtained by plotting the fluorescence vs. the logarithm of the progressively diluted concentrations of HscB (Figure 3A). The data obtained by different values of light emitting diode (LED) resulted in a dissociation constant (K_D_) of 10–30 μM assuming a 1:1 stoichiometry. These affinities are low but comparable to those observed for other components of the *isc* machine (Prischi et al., 2010b) and, more importantly, to that observed for the IscU/HscB interaction (~10 μM, Hoff et al., 2000). To confirm the interaction with an independent method, we used chemical cross-linking, a technique able to capture weak or transient protein-protein interactions (Watson et al., 2012). We added bis[sulfosuccinimidyl]suberate, a cross-linking agent that reacts with primary amino groups, to a mixture containing HscB and IscS. SDS-PAGE revealed the presence of a new species at around 65 KDa that corresponds to HscB-IscS covalently bound (Figure 3B).

We mapped the residues of HscB involved by ^15^N HSQC NMR experiments recorded on a ^15^N labeled HscB sample titrated with unlabeled IscS up to a 1:2 HscB:IscS molar ratio. At a 1:1 IscS/HscB molar ratio, many peaks, amongst which those from the J-domain, do not shift (Figure 3C). This suggests that IscS has a minimal effect on these regions of HscB. Other resonances (L22, H63, Q95, R99, E104, E106, E111, I118, K119, M124, L157, A161, and D170) have small but detectable chemical shift changes indicative of environmental changes in the presence of IscS. These residues are mainly located on the C-terminus and map on the surface facing the J-domain. A third set of residues (I105, I118, F125, H129, L131, L136, D139, L151, D155, R158, E162, and K167) broaden significantly. At the end of the titration, the HscB spectrum disappears almost completely as expected for the formation of a complex with a molecular weight of 130 kDa (assuming a 1:1 stoichiometry). Complexes of this size are not usually observable without deuteration because their large correlation times (τ_c_) causes excessive line broadening of the resonances. To ensure that these variations involve a direct interaction rather than a conformational change which could propagate far from the surface of contact, we performed cross saturation experiments (Takahashi et al., 2000) using deuterated HscB. The following residues were observed to have an effect: L22, Q49, Q56, A80, Q95, D110, E111, K119, S160, Q163, E166, D170, and F171. Most of these residues are close in space and located in the C-terminus of the approximately L-shaped HscB structure and form an exposed patch (Figure 3D). We validated the surface of interaction on HscB by designing two acid-to-base mutants which can be expected to affect binding (HscB_E111K/E115K and HscB_E165K/E166K). Their circular dichroism spectra are superposable on that of the wild-type proving that they retain the fold (Figure S1). The enzymatic rates in the presence of the individual mutants led to effects that approach the behavior observed in the absence of HscB.

Overall, these results validate the binding interface on HscB. We can thus conclude that there is a weak but specific binding between HscB and IscS.

### HscB competes for the CyaY binding site and is compatible with IscU binding

**Figure 4:**
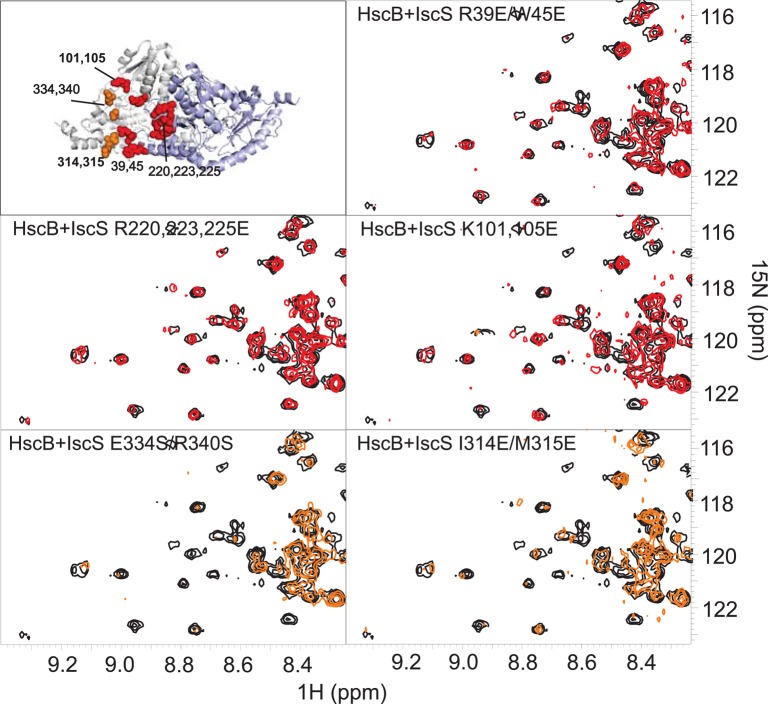
**Identification of the surface of interaction on IscS by mutagenesis**. Position of the mutations on the structure of IscS (PDB code: 3LVL) and HSQC-NMR spectra of 15N-labeled HscB 100 μM (black) and in the presence of the IscS mutants (1:1). Spectra in red are not affected, spectra in orange show the disappearance of few peaks because of the formation of a complex IscS:HscB. The same color coding is used in the structure panel.

In turn, we tested the ability of *ad hoc* designed IscS mutants (IscS_R39E/W45E, IscS_K101E/K105E, IscS_R220E/R223E/R225E, IscS_I314E/M315E, and IscS_E334S/R340S) which affect different regions of the protein to identify the surface of IscS interacting with HscB. Circular dichroism supports that the mutants are all correctly folded as expected from their location in exposed regions of the enzyme (Prischi et al., 2010b). Among them, IscS_R220E/R223E/R225E, IscS_R39E/W45E, and IscS_K101E/K105E leave mostly unaffected the spectrum of ^15^N HscB (Figure 4). This means that mutations of these residues results in complete or partial abolishment of binding. Conversely, the spectrum is affected by IscS_I314E/M315E and IscS_E334S/R340S titration comparably to wild-type, indicating that these residues are not involved in the interaction. A similar behavior has been reported for the frataxin ortholog CyaY (Prischi et al., 2010b) and for Fdx (Yan et al., 2013) which also form complexes with IscS with micromolar affinities. These results thus suggest that HscB binds IscS in a site overlapping with that observed for these proteins.

**Figure 5:**
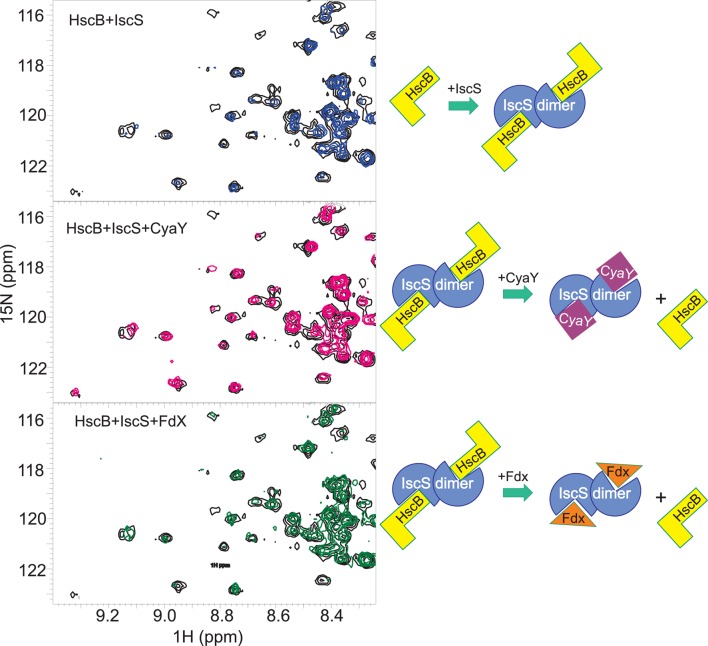
**Competition experiments of HscB with CyaY and Fdx. (Top)** Superposition of the HSQC spectra of 100 μM free 15N-labeled HscB (black) and in the complex with unlabelled IscS 1:1 (blue). **(Middle)** HSQC spectra of 100 μM 15N-labeled HscB in complex with IscS with the addition of CyaY (1:1:3). **(Bottom)** HSQC spectra of 100 μM 15N-labeled HscB in the presence of IscS and apo-Fdx (1:1:3). The experiments are summarized schematically on the right.

To validate the hypothesis, we titrated a sample containing the HscB/IscS complex with CyaY (up to a 1:1:3 molar ratio) or Fdx (up to a ratio 1:1:3). We observed that introduction of CyaY causes the progressive reappearance of the spectrum of HscB, consistent with almost complete displacement of IscS at 1:1:1 (Figure 5, top and middle panels). Titration of the HscB/IscS complex with Fdx also regenerates the spectrum of HscB but at a higher molar ratio: at a 1:1:3 ratio of IscS/HscB/Fdx, the spectrum of HscB is only partially restored (Figure 5, top and bottom panels). This is in agreement with the relative binding affinities (Prischi et al., 2010b; Yan et al., 2013) and confirms that the interaction between IscS and HscB involves the same site which hosts CyaY and Fdx. The site involves a cleft formed between the two protomers of the IscS dimer and contains PLP and the catalytic center (Cupp-Vickery et al., 2003).

**Figure 6:**
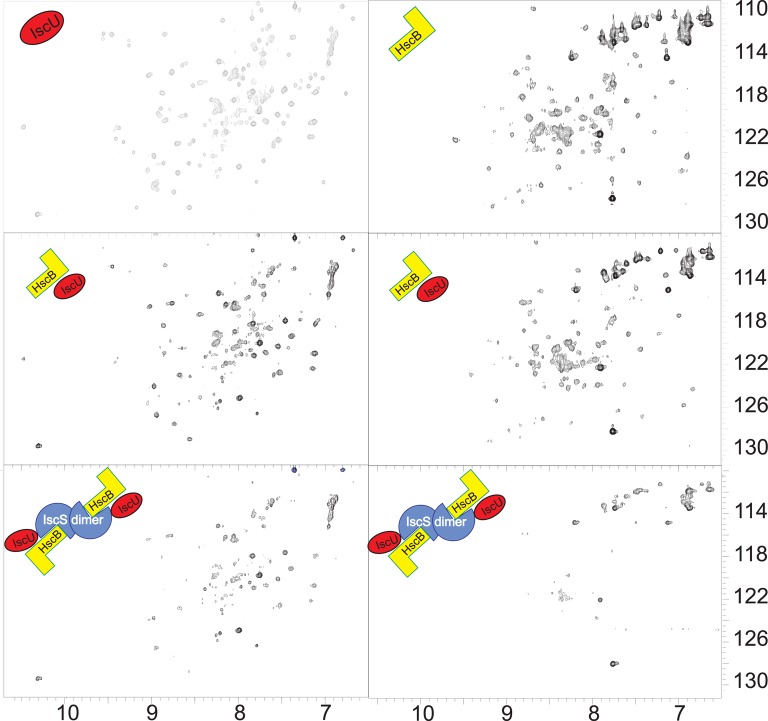
**IscU does not compete with IscS binding. Left:** Comparison of the spectra of ^15^N-labeled IscU **(top)**, the same but after addition of HscB 1:0.4 **(middle)** and after the further addition of IscS 1:0.4:0.4 **(bottom)**. **Right:** Comparison of the spectra of ^15^N-labeled HscB **(top)**, the same but after addition of IscU 1:1 **(middle)** and after the further addition of IscS 1:1:1 **(bottom)**.

Finally, we checked if the HscB/IscS complex is compatible or mutually exclusive with IscU binding. We added unlabeled HscB to ^15^N labeled IscU (1:0.4 IscU:HscB) and observed the typical chemical shifts expected for binding (Figure 6, **Left**). We then added unlabeled IscS (1:0.4:0.4) and observed further disappearance of the spectrum. Since this could simply mean a displacement of HscB, we also titrated labeled HscB with unlabeled IscU (1:1), to which we added unlabeled IscS (1:1:1). Also in this case the spectrum disappears almost completely (Figure 6, **Right**). Titration of HscB with IscS first and then with IscU led to the same result (data not shown). These results conclusively indicate that interaction between HscB and IscU is compatible with IscS binding, a hypothesis not previously considered.

## Discussion

In the last 15 years, increasing efforts have been dedicated to understand the mechanism of iron-sulfur cluster biogenesis because this is an essential machine common to all organisms which increasingly appears to have an important role in human disease. Precious new information was obtained by studies in cellular or animal models, such as yeast, drosophila (Kondapalli et al., 2008) and mice (Pandey et al., 2015). *In vitro* studies, which work with a well-defined number of highly purified components, have nevertheless remained essential to clarify the role of each of the players and understand the chemistry which drives cluster formation. A main difficulty in analysing the machine *in vitro* is, however, the complexity of the multi-component system in which each protein can further be in several possible states (oxidized/reduced, cluster-loaded/cluster-free, etc.). The additional presence of iron cations, nucleotides, and cysteine further complicate the picture. This increases the number of possible interactions combinatorially.

Probably for this reason, there are still relatively few studies that address the role of HscB and HscA in the *isc* machine. Chandramouli and Johnson had showed that HscA and HscB from *A. vinelandii* stimulate [2Fe-2S] cluster transfer from IscU to apo-Fdx in an ATP-dependent reaction (Chandramouli and Johnson, 2006). These researchers, however, probed only the second step of the reaction recording formation of the cluster on Fdx in the absence of the desulfurase. More recently, a study of the role of HscB and HscA from *E. coli* was carried out by Iametti and coworkers who showed an inhibitory effect of the HscA/HscB/ATP system when the cluster is formed on IscU which is independent from the source of sulfur (chemical or enzymatic) (Iametti et al., 2015). They also used Fdx as the reporter for chemical cluster formation and observed an enhancement. The effect was explained by the well described interaction between IscU and the chaperones (Hoff et al., 2000; Fuzery et al., 2008).

We started our study to explore the effect further. We first explored the multidimensional space of the *isc* interactome keeping the minimal number of components (IscA and IscR are thought to enter in the picture as independent players) and optimizing the relative concentrations within ranges of concentration biologically reasonable. We then introduced the components individually to identify their contributions. We observed that HscB, alone and in the co-presence of HscA, can have detrimental effects on the rate of cluster formation depending on their range of concentrations. This is at strong variance with what could have been expected for chaperones intended as “helper of folding” (Silberg and Vickery, 2000; Silberg et al., 2004). We could demonstrate that, in addition to be related to the interaction between IscU and HscB as previously interpreted (Iametti et al., 2015), the inhibitory influence of HscB acts also on the first step of FeS cluster biosynthesis interfering and slowing down cysteine desulfuration.

We reasoned that the main difference between our and previous studies (Iametti et al., 2015) was the absence of IscS in the latter and explored the possibility of a previously unidentified direct interaction between the two proteins. Using NMR and microscale thermophoresis, two techniques which can detect interactions with high sensitivity, we could prove that HscB and IscS interact together and that the surface of interaction involves the long stem of the L-shaped structure of HscB and the cavity of IscS close to the active site. The biological relevance of the interaction cannot easily be doubted since the affinity is comparable with that of by now established interaction with IscU (both K_D_s are around 10 μM; Hoff et al., 2000). The surface of interaction involves the region of IscS in the interface between the two protomers and the active site. This surface has already been involved in the interaction with other components of the *isc* machine which bind with similar weak affinity (Prischi et al., 2010b; Yan et al., 2013).

Our results thus suggest the possibility that IscS acts as the template and mediates the interaction of the chaperones with its preferential partner IscU. An IscS-mediated interaction with IscU was also observed for the bacterial CyaY which does not interact directly with IscU at variance with the longer eukaryotic ortholog (Layer et al., 2006; Adinolfi et al., 2009). Our unexpected results may change completely our perspective of the events involved in the *isc* machine: the whole scene of action for FeS cluster formation seems now to gravitate around IscS rather than involving two different machines, one on IscS and the other on the two chaperones, as previously suggested. It also proposes a potentially different role of the two chaperones and possibly of all chaperones: they are not just passive players able to “help” folding but more active components of the cellular machine. A similar view was also put forward in a recent comprehensive review (Finka et al., 2016) in which it is proposed that all chaperones are unfoldases. This hypothesis could explain the inhibitory effect of HscB and HscB/HscA on cluster formation and on desulfuration and would be consistent with the structure of the complex of HscA with a L^99^PPVK^103^ peptide from IscU, where the peptide, which forms a tight turn in full-length IscU, is fully extended (Cupp-Vickery et al., 2004). It would however, be at variance with evidence suggesting that HscB binds preferentially the structured state of IscU (Kim et al., 2012) and leave open the exact role of HscB on IscS.

Interestingly the proposed binding sites for IscU on HscB involve residues 92, 93, and 153 (Fuzery et al., 2011) which are in the same region which binds to IscS but on the opposite phase of the helical hairpin. Concomitant binding of HscB with IscU and IscS is thus possible, according to what we observe experimentally.

**Figure 7:**
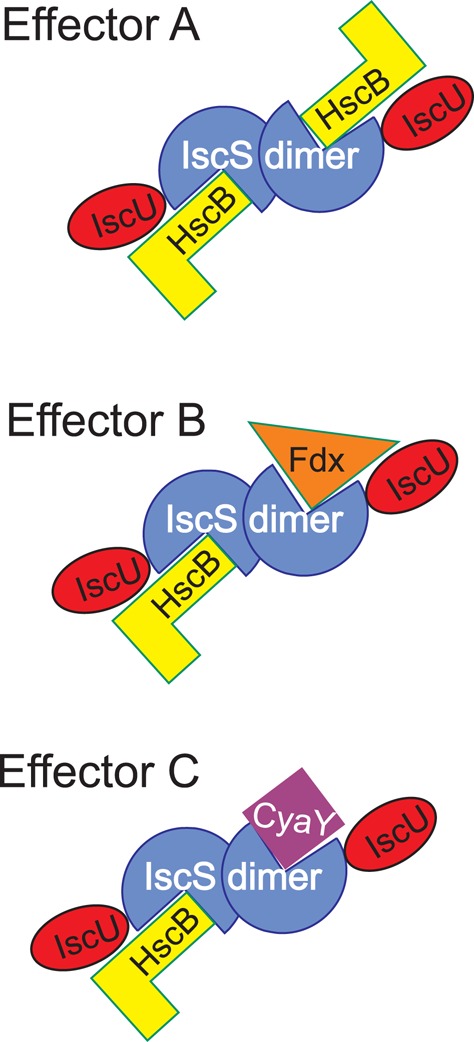
**Scheme to illustrate the possible regulation of the IscS interactions**. Different effectors may signal which components are bound to IscS at any time point keeping in mind that IscS is a dimer. The possibility of occupying two independent binding sites also makes room for the possibility that allosteric mechanisms may operate.

Another aspect that our research reveals is how the multiple interactions of IscS with very different proteins involve the same surface of interaction. This will require a tight regulation on which specific protein is bound at the different time points. A possible way to explain our findings is to remember that IscS is a dimer (Figure 7). This means that, in the presence of different suitable effectors, different interactions may in principle be possible on the two IscS protomers leading to a complex but efficient regulation according to an allosteric mechanism.

While much more needs to be done to completely unveil our structural and functional understanding of the FeS cluster biogenesis, our studies add a new tessera to the comprehension of the *isc* machine and its regulation and open new avenues to our perception of the role of chaperones.

## Materials and methods

### Protein purification

Proteins were subcloned by PCR from bacterial genomic DNA and individually expressed from pET-derived plasmid vectors as fusion proteins with His-tagged glutathione-Stransferase (GST) and a cleavage site for Tobacco Etch Virus (TEV) protease. They were expressed and purified from *E. coli* strain BL21(DE3) as previously described (Adinolfi et al., 2002; Prischi et al., 2010a). Cells were inoculated at 37°C in LB medium with kanamycin (30 mg/l), induced for overnight at 18°C by the addition of 0.5 mM isopropyl β-D-thiogalactopyranoside (IPTG) after the cultures reached an optical density (OD) at 600 nm of 0.6–0.8. Cell pellets were centrifuged, resuspended in a lysis buffer (20 mM Tris-HCl pH 8, 150 mM NaCl, 10 mM imidazole, Igepal, DNAse I, lysozyme, antiprotease, 1 mM TCEP) and frozen. Cell pellets were then thawed and sonicated and centrifuged. Proteins were purified by affinity chromatography (using Ni-nitrilotriacetic agarose), eluted and cleaved overnight from His, GST-tag by TEV protease dialysing in 20 mM Tris-HCl pH 8, 150 mM NaCl, 1 mM DTT. Further purification was carried out by gel filtration chromatography on a 16/60 Superdex G75 column. Samples were eluted in 20 mM Tris-HCl pH 8.0, 150 mM NaCl, and 1 mM TCEP and monitoring absorbance at 280 nm. Protein concentration was determined by UV spectroscopy. Protein purity was checked by SDS-PAGE after each step of the purification. ^15^N labeled samples for NMR measurements were grown in minimal medium using (^15^NH_4_)_2_SO_4_ as the sole source of nitrogen while ^15^N,^2^H labeled samples were grown in minimal medium containing D_2_O and using (^15^NH_4_)_2_SO_4_.

### Reconstitution experiments

Enzymatic cluster formation was achieved under strict anaerobic conditions in a Belle chamber kept under nitrogen atmosphere. The reaction was followed by absorbance spectroscopy using a Cary 50 Bio Spectrophotometer (Varian). Absorbance variations at 458 or 406 nm were measured as a function of time. A solution of 50 μM of apoFdx or 35 μM aconitase was incubated in sealed cuvettes typically using 3 mM DTT, IscU, HscA, HscB, 1 μM IscS, and 25 μM Fe(NH_4_)_2_(SO_4_)_2_ for 30 min in 20 mM Tris-HCl pH 8 and 150 mM NaCl. The reaction was initiated by adding 250 μM of the substrate L-cysteine and when specified 150 μM ATP. Each experiment was repeated at least 3 times on different batches of proteins and by two different researchers. To simplify the analysis, we took the initial slopes of the curves (absorbance vs. time) to qualitatively compare the time courses although some curves have a lag phase which likely reflects diffusion of the components.

### MST

MST measurements were performed using a NanoTemper Monolith NT.115 instrument. IscS samples were labeled with the amine-reactive dye NT-647 using the Monolith NT.115 Protein Labeling Kit RED-NHS. The reaction mixture was incubated overnight at 4°C. Labeling levels (in the range of 0.67 dye molecules per IscS) were determined using ε_280_(IscS) 41,370 M^−1^cm^−1^ and ε_650_(dye) 250,000 M^−1^cm^−1^. HscB was dissolved to a final concentration of 600 μM in a buffer containing 20 mM Tris-HCl pH 8, 150 mM NaCl, 0.5 mM TCEP, 0.05% Tween-20 and labeled IscS at a concentration of 52 nM. The stock solution was then serially diluted to 1:1 using the same buffer to give 12 working solutions with different HscB concentration but the same fluorophore concentration. The solutions were then loaded into hydrophilic capillaries and MST measurement were made at 25°C using 40–60% light-emitting diode power and 17% of infrared-laser power. Each measurement was repeated at least 3 times.

### Mass spectrometry

Alanine formation was obtained with 1 μM IscS, 3 mM DTT, and 250 μM cysteine in 20 mM Tris-HCl at pH 8 and 150 mM NaCl. When necessary, 3 μM HscB was added. The reaction was stopped with a 150 μl mixture acetic acid (0.1%)/acetonitrile (100%) after 0, 10, 20, 40, and 60 min. Determination of alanine and cysteine was performed by HR-LCMS using a Thermo Accela Pump and Pal Autosampler coupled to a Thermo EXactive. Chromatographic separation was performed on a ODS Hypersil 150 × 2.1 mm 3u column maintained at room temperature. A 10 min gradient was employed using a mobile phase A with 0.1% formic acid in water and a mobile phase B in 0.1% formic acid in acetonitrile.

### NMR spectroscopy

NMR spectra were acquired on Bruker AVANCE spectrometers operating at 600, 700, and 800 MHz proton frequencies. Typically, measurements were carried out at 298 K in 20 mM Tris-HCl pH 8, 150 mM NaCl, and 2 mM DTT using a 0.1 mM uniformly ^15^N-enriched protein. Water suppression was achieved by using WATERGATE and HSQC experiments were recorded. The spectra were processed by using the NMRPipe program and analyzed by Ccpnmr. Cross saturation experiments were carried out on ^2^H,^15^N-labeled HscB sample and using TROSY detection. Spectral assignment of HscB was based on the BMRB deposition.

### Cross-linking

A mixture of 5 μM IscS and 30 μM HscB was prepared in PBS buffer. Bis[sulfosuccinimidyl]suberate was added to the protein sample to a final concentration of 1.3 mM. The reaction mixture was incubated at room temperature for 30 min and quenched by adding Tris-HCl at pH 8 to a final concentration of 20 mM. The quenching reaction was incubated at room temperature for 15 min. SDS-PAGE was run to observe the cross-linked species.

## Author contributions

RP carried out most experiments, RY assisted and provided specific valuable expertise. SA and AP conceived the research. RP and AP wrote the paper.

### Conflict of interest statement

The authors declare that the research was conducted in the absence of any commercial or financial relationships that could be construed as a potential conflict of interest.
