# Supplementary figures and images for: A New Tessera into the Interactome of the isc Operon: A Novel Interaction between HscB and IscS
Source: Front Mol Biosci. 2016 Sep 27;3:48. doi: 10.3389/fmolb.2016.00048 (PMC5037179; doi:10.3389/fmolb.2016.00048)

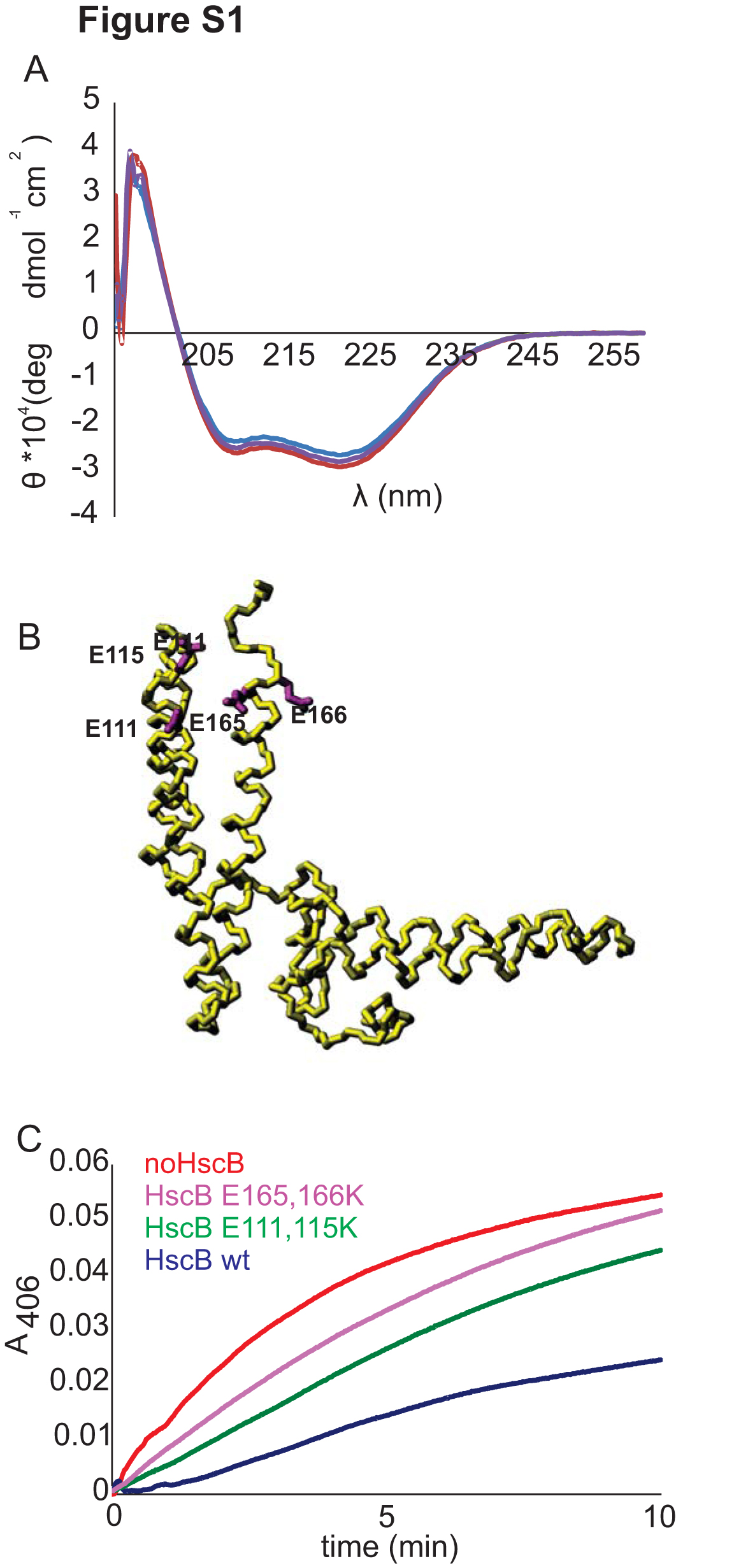

Supplement: Supplementary file 1 [file Image1.jpg]
